# Significant myopic shift over time: Sixteen-year trends in overall refraction and age of myopia onset among Chinese children, with a focus on ages 4-6 years

**DOI:** 10.7189/jogh.13.04144

**Published:** 2023-11-09

**Authors:** Zhi Chen, Dantong Gu, Bingjie Wang, Pauline Kang, Kathleen Watt, Zuyao Yang, Xingtao Zhou

**Affiliations:** 1 Eye Institute and Department of Ophthalmology, Eye & ENT Hospital, Fudan University, Shanghai, China; 2 Clinical Research and Achievement Translation Center, Eye & ENT Hospital, Fudan University, Shanghai, China; 3 NHC Key Laboratory of Myopia (Fudan University); Key Laboratory of Myopia, Chinese Academy of Medical Sciences, Shanghai, China; 4 Shanghai Research Center of Ophthalmology and Optometry, Shanghai, 200031, China; 5 Shanghai Engineering Research Center of Laser and Autostereoscopic 3D for Vision Care, Shanghai, China; 6 School of Optometry and Vision Science, University of New South Wales, Australia; 7 Division of Epidemiology, JC School of Public Health and Primary Care, The Chinese University of Hong Kong, Hong Kong SAR, China

## Abstract

**Background:**

Myopia or near-sightedness is a major cause of blindness in China and typically develops between the ages of 6-12 years. We aimed to investigate the change in refractive error and the age of myopia onset in Chinese children from 2005 to 2021.

**Methods:**

We first conducted a series of cross-sectional studies to determine the refractive states and the age of myopia onset over time, after which we analysed longitudinal data to investigate the dose-response relationship between hyperopic reserve and future risk of myopia. The analysis was based on the refraction data of children aged 4-18 years who visited the Fudan University Eye and Ear, Nose, and Throat (FUEENT) Hospital, a large tertiary hospital in Shanghai, China, for eye examinations between 2005 and 2021. We examined the prevalence of hyperopia (spherical equivalent refractive error (SERE) >0.75D), pre-myopia (-0.50D < SERE ≤ 0.75D), and myopia (SERE ≤-0.50D), the average SERE for each age group at the initial visit, the average age of myopia onset, and the safety threshold of hyperopic reserve against myopia onset.

**Results:**

We included 870 372 eligible patients aged 4-18 years who attended examination between 2005 and 2021, 567 893 (65.2%) of whom were myopic at their initial visit to FUEENT. The mean SERE decreased in most (n/N = 14/15) of the age groups over the 16 calendar years, with a mean SERE for the whole cohort decreasing from -1.01D (standard deviation (SD) = 3.46D) in 2005 to -1.30D (SD = 3.11D) in 2021. The prevalence of pre-myopia increased over the 16 years (*P* < 0.001), while those of myopia and hyperopia remained largely stable (both *P* > 0.05). We observed a significant decrease in the prevalence of hyperopia (2005: 65.4% vs 2021: 51.1%; *P* < 0.001) and a significant increase in the prevalence of pre-myopia (2005: 19.0% vs 2021: 26.5%; *P* < 0.001) and myopia (2005: 15.6% vs 2021: 22.4%; *P* < 0.001) in children aged 4-6 years. We found an earlier myopia onset over time, with the mean age of onset decreasing from 10.6 years in 2005 to 7.6 years in 2021 (*P* < 0.001). Children with a hyperopic reserve of less than 1.50D were at increased risk of developing myopia during a median follow-up of 1.3 years.

**Conclusions:**

We found an overall myopic shift in SERE in Chinese children aged 4-18 years over the past 16 years, particularly in those aged 4-6 years. The mean age of myopia onset decreased by three years over the same period. The “safety threshold” of hyperopic reserve we identified may help target the high-risk population for early prevention.

Myopia is a refractive state where the ocular axial length is too long for its refractive power [1]. Its global prevalence was estimated to have increased from 28.3% to 34.0% between 2010 and 2020 [2], especially in East Asian countries including China [3]. According to a recent Chinese government official report, the overall prevalence of myopia in Chinese primary, secondary, and high school students was 36.0%, 71.6% and 81.0%, respectively [4].

Children who develop myopia experience accelerated axial length elongation up to 2-3 years before myopia onset [5,6], which would lead to a myopic shift in their refractive status away from the age-normal hyperopia [7]. Consequently, the 2019 International Myopia Institute defined “pre-myopia” in their white paper report as a “refractive state of an eye of ≤0.75D and >-0.50D” in children, where a combination of baseline refraction, age, and other quantiﬁable risk factors can help predict the likelihood of the future development of myopia to inform preventative interventions.

Myopia typically develops between 6 and 12 years [8], with earlier onset age associated with greater risk of high myopia [9] and associated ocular pathologies [10] in the future. Characterising temporal changes in hyperopic and pre-myopic refractive status in children aged 4 to 6 years may improve our understanding of the overall trend of myopia onset and prevalence over time. However, large-scale refractive data in these younger children is lacking in current literature. We therefore aimed to investigate changes in the refractive states of Chinese children aged 4-18 years over time, with a particular focus on younger children aged between 4-6 years, using a clinical sample from a tertiary eye specialty hospital in Shanghai, China. We also aimed to investigate the change in age of myopia onset over time and the dose-response relationship between hyperopic reserve and future risk of developing myopia.

## METHODS

We first conducted a series of cross-sectional data to determine the refractive states and the age of myopia onset over time, after which we adopted a longitudinal study design to assess the dose-response relationship between hyperopic reserve and future risk of myopia in a subgroup of the participants who did not have myopia at baseline and had at least one follow-up visit to determine whether they had progressed to myopia. We conducted the study at Fudan University Eye and Ear, Nose, and Throat (FUEENT) Hospital in Shanghai, China, a tertiary specialty hospital with annual outpatient visits exceeding two million. We received approval from the hospital’s ethics committee (No. 2022145).

We included all eligible patients aged 4-18 years, based on the following inclusion and exclusion criteria:

1. Spherical equivalent refractive error (SERE) and prevalence of different refractive states over time. Inclusion criteria – at least one set of refractive data available to determine the patient’s refractive state, with the initial visit used for analysis;
2. Age of myopia onset over time. Inclusion criteria – at least two sets of refractive data available, with one for the hyperopic or pre-myopic state and another in the myopic state on a subsequent visit;
3. Dose-response relationship between hyperopic reserve and future risk of myopia. Inclusion criteria – at least two sets of refractive data available, with at least one set showing hyperopia or pre-myopia, regardless of refractive states observed in subsequent visits.

We excluded patients with ocular pathologies that may impact refractive development in children, including congenital cataracts, strabismus, glaucoma, prescription of low-concentration atropine eye drops, and prescription of optical modalities other than single vision lenses (e.g. multi-focal spectacles or contact lenses, orthokeratology contact lenses).

We retrieved data on patients’ gender, age, and refractive data including the spherical power, cylindrical power, cylindrical axis, best corrected distance visual acuity, and cycloplegic agent used for examination from the hospital’s electronic medical record system from 2005 to 2021. We also collected outstanding medical history, including ocular pathologies, concurrent medication use, and optical treatment modality. We included all patients who met the inclusion criteria within the electronic medical history system throughout the 2005-2021 period; however, data from 2013 and 2014 were not available due to a system update and catastrophic loss of backup data.

### Subjective refraction

We used cycloplegia in all children aged ≤12 years and optionally used in children >12 years before refraction. Cycloplegia was achieved by administering 0.5% tropicamide eye drops five times in a row in five-minute intervals. Subjective refraction was measured 30 minutes after the last eye drop. Hospital-based opticians used the standardised maximum plus for best visual acuity method in all subjective refraction procedures.

### Definitions

#### Refractive state

We defined SERE as *sphere* + *cylinder*/*2* and used the less hyperopic eye to define the refractive state. We defined a SERE >0.75D as hyperopia, -0.50D < SERE ≤ 0.75D as pre-myopia, and ≤-0.50D as myopia.

#### Myopia onset

We defined myopia onset as a shift in the refractive state from hyperopia or pre-myopia to myopia in two consecutive visits, with the age of the child at the latter visit defined as myopia onset age.

### Statistical analyses

We calculated the mean SERE and its standard deviation (SD) for each age group and for all children as a whole, stratified by calendar years, which were then compared across groups using *t*-tests. We expressed the number of people with different refractive states as an absolute value (percentage) and compared them using χ*^2^* tests. We used the Mann-Kendall test to determine whether there was a linear monotonic trend in the mean SERE, proportion of different refractive states, and mean age of myopia onset from 2005 to 2021.

We used multivariate Cox regression models to assess the dose-response relationship between SERE and the onset of myopia, adjusting for age and gender. To investigate the safety threshold of SERE below which the risk of myopia at subsequent visits increased significantly, we used restricted cubic splines (RCS) with four knots at the 5^th^, 35^th^, 65^th^, and 95^th^ percentiles to estimate the adjusted hazard ratios (HR) and 95% confidence intervals (CIs). We tested for potential nonlinearity by using a likelihood ratio test comparing the model with only a linear term against the model with linear and cubic spline terms. We regarded a SERE for which the HR was 1 as the safety threshold. We also employed a linear Cox model to estimate the HR for myopia corresponding to an increase of 0.1D in SERE below and above the threshold, respectively. We performed all statistical analyses in R, version 4.2.2 (R Core Team, Vienna, Austria) and Python, version 3.7 (Python Software Foundation, Delaware, USA), setting the significance level set to *P* < 0.05 (two-sided).

## RESULTS

**Figure 1:**
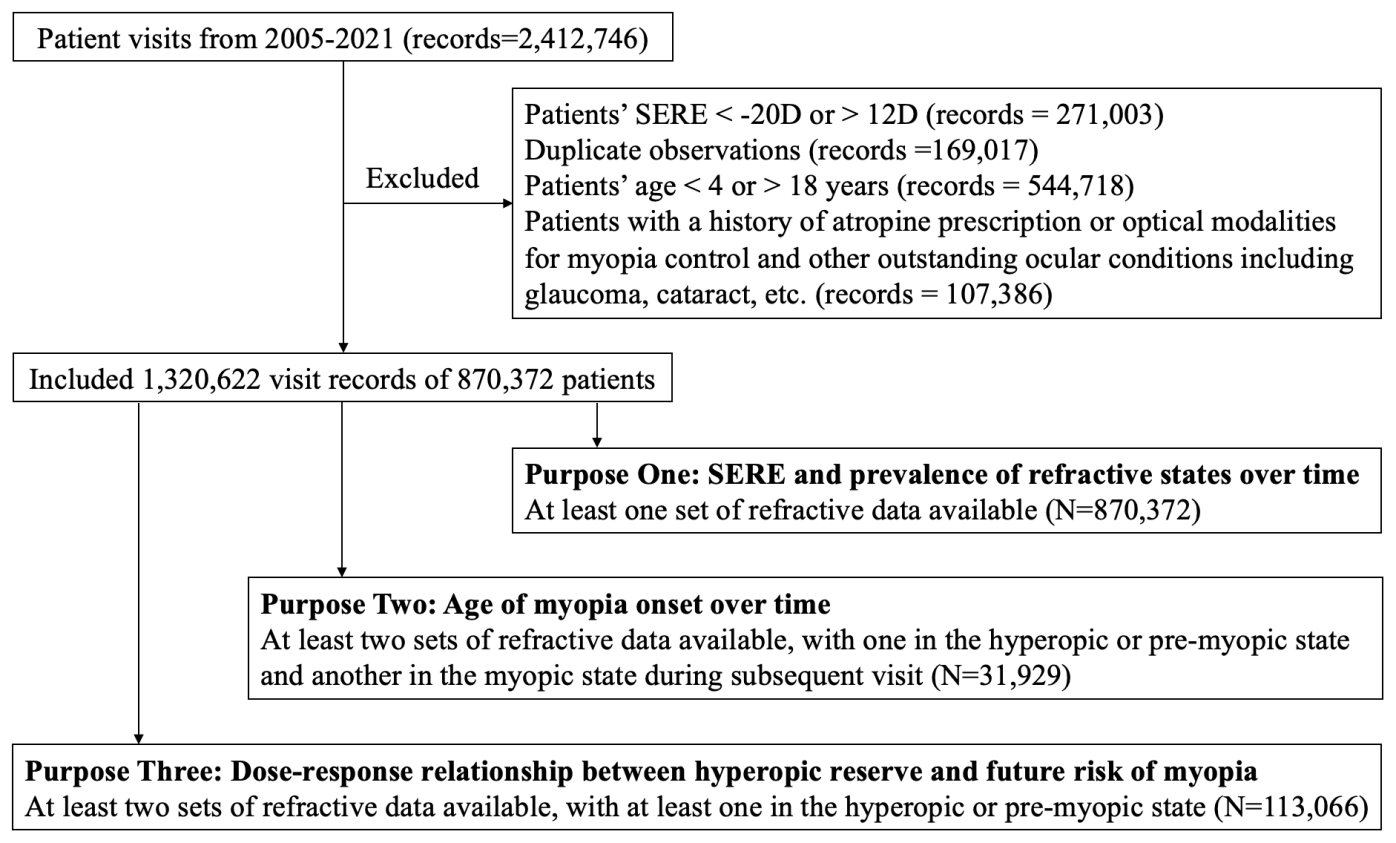
Flowchart of participants recruitment.

We included 870 372 eligible patients aged 4-18 years who attended examinations between 2005 and 2021, 567 893 (65.2%) of whom were myopic at their initial visit to FUEENT (**Figure 1**).

### SERE and prevalence of refractive states over time

**Figure 2:**
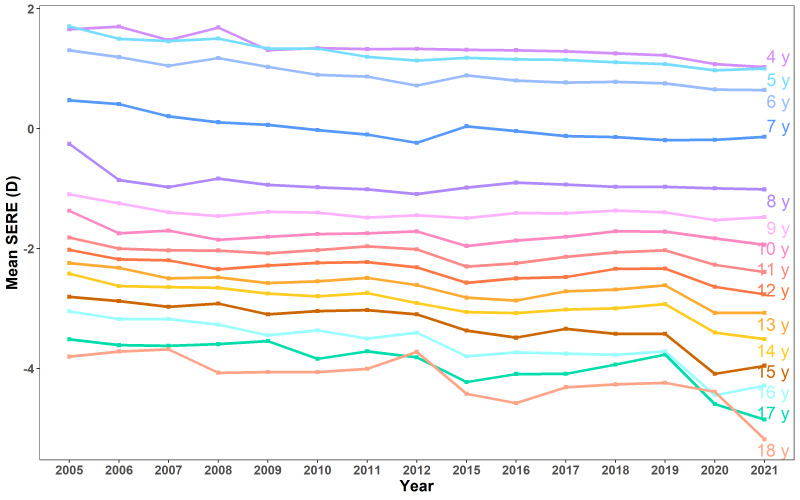
The change in mean SERE of children aged 4-18 years from 2005 to 2021.

We observed a significant shift in refraction towards higher myopia over time across most of the age groups (*P* < 0.05 except for the 10-year-old group), with the mean SERE for the whole cohort decreasing from -1.01 (SD = 3.46D) in 2005 to -1.30 (SD = 3.11D) in 2021 (**Figure 2** and Table S1 in the **Online Supplementary Document**).

**Figure 3:**
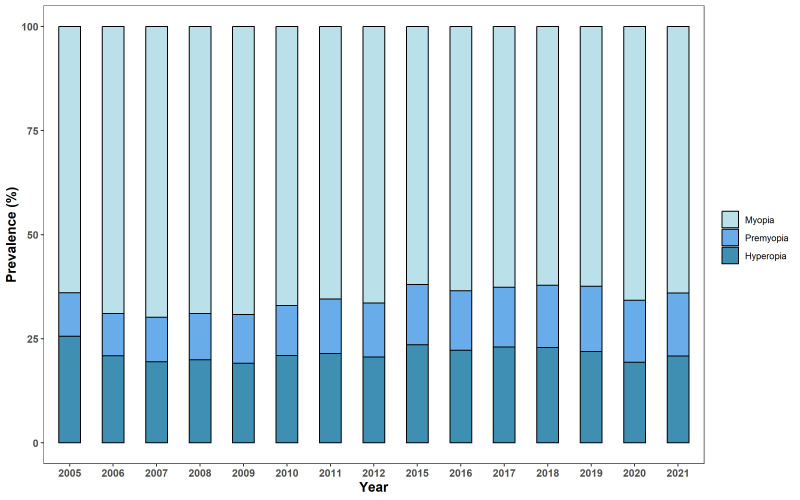
Prevalence of myopia, pre-myopia, and hyperopia in children aged 4-18 years from 2005 to 2021.

The prevalence of pre-myopia in children aged 4-18 years from 2005 to 2021 significantly increased over time (*P* < 0.001), while the prevalence of myopia (*P* = 0.11) and hyperopia (*P* = 0.84) remained relatively stable over time (**Figure 3** and Figure S1 in the **Online Supplementary Document**). Further analysis for children aged 4-6 years showed a significant decrease in the prevalence of hyperopia (2005: 65.4% vs 2021: 51.1%; *P* < 0.001) and a significant increase in the prevalence of pre-myopia (2005: 19.0% vs 2021: 26.5%; *P* < 0.001) and myopia (2005: 15.6% vs 2021: 22.4%; *P* < 0.001). The prevalence of high myopia (defined as SERE≤-6.00D) in children aged 16-18 years increased significantly over time (*P* < 0.001), from 15.7% in 2005 to 32.4% in 2021 (Figure S2 in the **Online Supplementary Document**).

### Age of myopia onset over time

**Figure 4:**
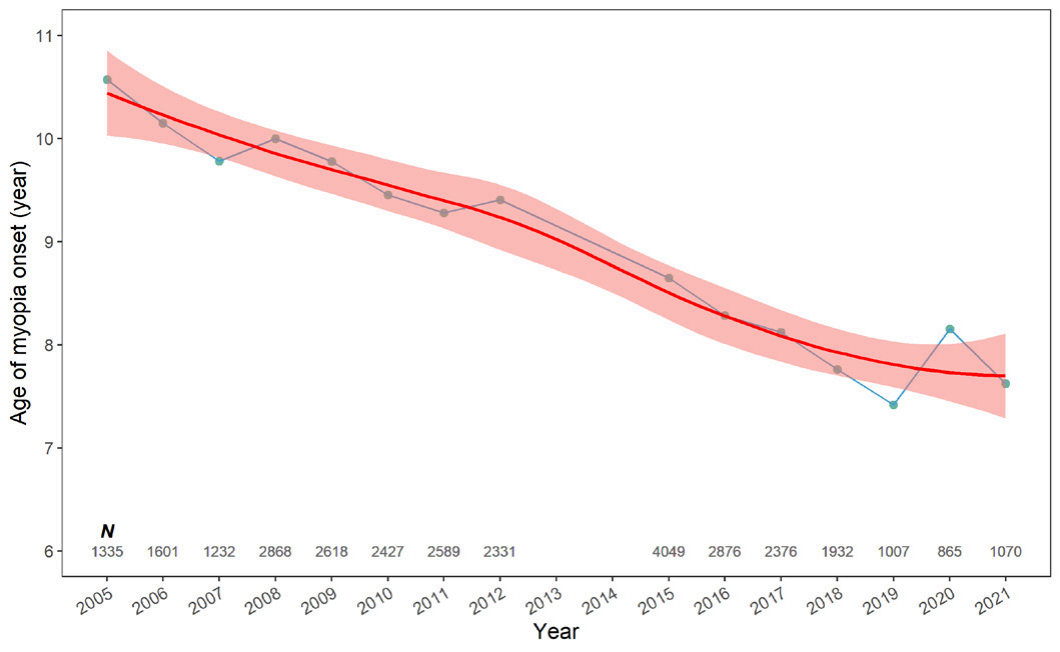
Age of myopia onset over time from 2005 to 2021. Data points represent mean age of myopia onset, with the redline representing the locally weighted regression smooth curve (LOESS) and the shaded area indicating the 95% confidence intervals.

Overall, 31 929 children experienced myopia onset between 2005 and 2021, with a mean follow-up frequency of 4.2 times. The age of myopia onset steadily decreased over time, from a mean of 10.6 years in 2005 to 7.6 years in 2021 (*P* < 0.001) (**Figure 4**).

### Dose-response relationship between hyperopic reserve and future risk of myopia

**Figure 5:**
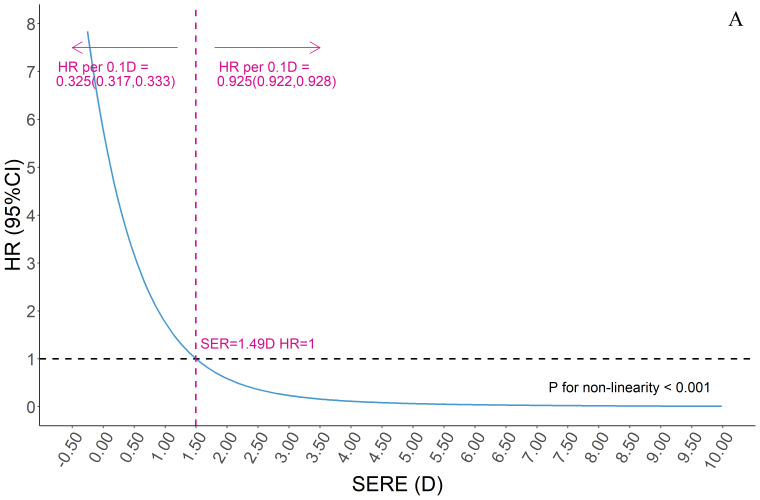
Dose-response relationship between hyperopic reserve and future risk of myopia. SERE – spherical equivalent refractive error. HR – hazard ratio.

The children included in this analysis (n = 113 066) were followed up for a median of 1.3 years, with 28.2% of them developing myopia during the period. The risk of myopia increased rapidly when SERE at the first visit was lower than +1.50 D (nonlinearity *P *< 0.001). We found a change in HR per 0.10D SERE of 0.325 (95% CI = 0.317-0.333) below +1.50D and 0.925 (95% CI = 0.922-0.928) above +1.50D (**Figure 5**).

## DISCUSSION

We aimed to investigate changes in the refractive state and age of myopia onset of Chinese children aged 4-18 years over a 16-year period (from 2005 to 2021) using a clinical sample from a tertiary specialty hospital in Shanghai, China. We found a significant myopic shift in SERE, particularly in those aged 4-6 years, and earlier myopia onset over time.

A previous study based on large-scale clinical data from China investigated prevalence of myopia in a population with a wide range of age (1 to 95 years) and showed that the mean age of myopia onset was 7.5 years in children; however, a significant proportion of its participants did not use cycloplegia before measurement of refractive error, which could have compromised the accuracy of refraction data. Additionally, that study did not analyse the trend of myopia onset over time [11].

Decrease in age of myopia onset from 10.6 years in 2005 to 7.6 years in 2021 (i.e. by three years) can significantly impact overall myopia and high myopia prevalence in the population. Earlier onset of myopia has been found to predict greater risk of future high myopia in several studies [9,12,13]. This is likely due to faster myopia progression in younger children. For example, a previous study conducted in children of Asian ethnicity estimated that myopia progressed by a mean of 0.90D per year at seven years of age vs 0.46D per year at 14 years of age [14]. Higher myopia is associated with greater risk of posterior ocular pathologies such as myopic macular degeneration, a leading cause of blindness in East Asia [15]. Therefore, an earlier onset of myopia could significantly increase the likelihood of future visual impairment [16].

We also found that the safety threshold of hyperopic reserve associated with a reduced likelihood of future myopia onset was approximately +1.50D in children, regardless of age and gender. A few previous studies have investigated this issue [17-19]. For example, in their Collaborative Longitudinal Evaluation of Ethnicity and Refractive Error (CLEERE) study, Zadnik et al. [17] found that the cut-off cycloplegic SERE with optimized sensitivity and specificity was approximately +0.75D in students enrolled in grades 1-3 (6-8 years old) that was protective against myopia incidence in their 8^th^ grade.

The discrepancy between their SERE cut-off findings and ours could be partly attributed to the different ethnicity compositions of participants. The CLEERE study enrolled a ethnically diverse cohort, with 36.2% of the study population being white and 13.7% being Asian American [17]. White children and Asian children experience different trajectories during their refractive development; axial length growth is typically faster in Asian children compared to their white counterparts, even in non-myopic children [20,21]. Ma et al. [19] recruited a similar ethnic population to our study and found that the best cut-off SERE for predicting four-year incident myopia was SERE ≤0.75D in children enrolled in grade 1-3 students (area under the curve = 0.84). However, they used a linear logistic regression model to determine the cut-off value for screening purposes, which differs from our nonlinear RCS model. Our result suggests that Asian children need a higher hyperopic “reserve” during emmetropisation to remain non-myopic later in life.

It is unclear what caused the myopic shift in the study population over the past 16 years. Although myopia prevalence rates are high in East Asian countries, they also increased in other regions globally [15]. This suggests that environmental factors may play a strong causal role in the development of myopia and that the impact is more pronounced in East Asian regions. Asian children typically experience heavier academic burdens at a younger age and have less outdoor activity time than their Western counterparts [22]. Increasing outdoor time has shown promise in decreasing myopia incidence in primary school children in clinical studies as well as epidemiological studies [23-28]. In contrast, coronavirus 2019 (COVID-19) lockdowns dramatically increased myopia incidence in children aged 6-8 years [29]. Limited outdoor activity and unprecedentedly increased screen time during e-learning may have been attributed to the surge in myopia prevalence in these young cohorts during lockdown periods [30]. To effectively mitigate the burden of future myopia on the individual and society, factors contributing to the earlier myopic shift in younger children aged 4-6 years need to be determined.

The strength of this study lies in its long time span, which enabled us to observe the trend over 16 years. However, our findings should be interpreted with caution. First, the selection bias arising from hospital-based database might have led to overestimation of the prevalence of myopia because hyperopic and pre-myopic children without visual problems are less likely to visit the hospital. Thus, the absolute values of SERE and proportions of refractive states from a single centre might not be generalisable to all Chinese children. However, the relative change of these parameters over time may mirror the trend in the general population. Second, only refraction data, age, and gender were available for analysis; biometric data such as axial length, crystalline lens power, and keratometry, however, were not available within the electronic medical system. We were thus unable to explore the trend of ocular biometric change over time in addition to refractive change, but this was not our primary objective in the first place.

## CONCLUSIONS

We observed an earlier myopia onset and an overall myopic shift in refraction in a clinical sample of Chinese children aged 4-18 years over the past 16 years from 2005 to 2021. The prevalence of hyperopia decreased and that of myopia and pre-myopia increased over time in children aged 4-6 years, while the prevalence of high myopia more than doubled in children aged 16 to 18 years. Preventive measures to mitigate the earlier myopia onset and increased prevalence of younger children with myopia are required to reduce the disease burden of myopia. The safety threshold of hyperopic reserve in children may predict future myopia onset and can be used as indicators to initiate preventative measures in clinical practice.

## Additional material


Online Supplementary Document
