# Supplementary material for: Significant myopic shift over time: Sixteen-year trends in overall refraction and age of myopia onset among Chinese children, with a focus on ages 4-6 years
Source: J Glob Health. 2023 Nov 9;13:04144. doi: 10.7189/jogh.13.04144 (PMC10630697; doi:10.7189/jogh.13.04144)
Supplement: Online Supplementary Document [file jogh-13-04144-s001.pdf]

Table S1. Spherical equivalent refractive error（SERE) of children aged 4 to 18 years during their initial visit to FUEENT from 2005 to 2021

Age (year)	2005	2006	2007	2008	2009	2010	2011	2012	2015	2016	2017	2018	2019	2020	2021
4	1.65	1.70	1.48	1.69	1.31	1.34	1.33	1.33	1.31	1.31	1.29	1.25	1.22	1.07	1.02
	(3.32)	(3.57)	(3.46)	(3.09)	(3.36)	(3.26)	(3.23)	(3.25)	(3.13)	(3.22)	(3.18)	(3.17)	(3.05)	(3.07)	(3.00)
5	1.71	1.50	1.46	1.50	1.33	1.33	1.20	1.13	1.18	1.15	1.15	1.11	1.07	0.97	1.00
	(3.25)	(3.01)	(3.07)	(2.81)	(2.81)	(2.89)	(2.75)	(2.77)	(2.99)	(2.94)	(2.74)	(2.63)	(2.62)	(2.81)	(2.77)
6	1.31	1.19	1.05	1.17	1.03	0.90	0.87	0.72	0.89	0.80	0.77	0.78	0.75	0.65	0.64
	(3.29)	(3.00)	(2.95)	(2.87)	(2.76)	(2.88)	(2.72)	(2.72)	(2.95)	(2.82)	(2.75)	(2.65)	(2.60)	(2.80)	(2.83)
7	0.47	0.41	0.21	0.11	0.06	-0.03	-0.10	-0.24	0.04	-0.04	-0.12	-0.14	-0.19	-0.19	-0.14
	(3.39)	(3.05)	(2.86)	(2.94)	(2.67)	(2.71)	(2.70)	(2.63)	(2.87)	(2.67)	(2.54)	(2.58)	(2.40)	(2.64)	(2.69)
8	-0.26	-0.86	-0.97	-0.83	-0.94	-0.98	-1.01	-1.09	-0.99	-0.90	-0.93	-0.97	-0.97	-1.00	-1.01
	(3.24)	(2.73)	(2.43)	(2.46)	(2.46)	(2.36)	(2.27)	(2.18)	(2.53)	(2.37)	(2.32)	(2.15)	(1.97)	(2.32)	(2.49)
9	-1.10	-1.25	-1.40	-1.46	-1.39	-1.40	-1.48	-1.45	-1.49	-1.41	-1.41	-1.37	-1.40	-1.53	-1.48
	(2.94)	(2.59)	(2.38)	(2.24)	(2.17)	(2.15)	(1.96)	(2.02)	(2.34)	(2.21)	(2.10)	(2.11)	(1.94)	(2.17)	(2.35)
10	-1.37	-1.75	-1.70	-1.85	-1.80	-1.76	-1.75	-1.71	-1.96	-1.87	-1.81	-1.71	-1.72	-1.83	-1.94
	(2.93)	(2.43)	(2.34)	(2.20)	(2.17)	(2.10)	(2.02)	(2.07)	(2.33)	(2.20)	(2.06)	(2.10)	(2.00)	(2.18)	(2.37)

11	-1.82	-2.00	-2.03	-2.03	-2.08	-2.03	-1.96	-2.01	-2.30	-2.25	-2.14	-2.06	-2.03	-2.27	-2.39
	(2.80)	(2.46)	(2.26)	(2.18)	(2.05)	(2.12)	(2.04)	(2.07)	(2.29)	(2.12)	(2.23)	(2.13)	(2.01)	(2.33)	(2.36)
12	-2.02	-2.18	-2.20	-2.35	-2.28	-2.24	-2.23	-2.31	-2.57	-2.50	-2.48	-2.34	-2.34	-2.64	-2.76
	(2.61)	(2.39)	(2.29)	(2.18)	(2.22)	(2.29)	(2.07)	(2.16)	(2.41)	(2.31)	(2.28)	(2.21)	(2.18)	(2.45)	(2.53)
13	-2.24	-2.32	-2.50	-2.48	-2.58	-2.55	-2.49	-2.61	-2.82	-2.87	-2.71	-2.68	-2.61	-3.07	-3.07
	(3.15)	(2.53)	(2.62)	(2.50)	(2.34)	(2.35)	(2.45)	(2.29)	(2.46)	(2.43)	(2.47)	(2.51)	(2.40)	(2.61)	(2.66)
14	-2.42	-2.63	-2.64	-2.65	-2.75	-2.79	-2.74	-2.91	-3.06	-3.08	-3.02	-3.00	-2.93	-3.40	-3.51
	(3.15)	(2.55)	(2.51)	(2.52)	(2.66)	(2.55)	(2.55)	(2.55)	(2.61)	(2.55)	(2.56)	(2.63)	(2.62)	(2.70)	(2.85)
15	-2.80	-2.88	-2.97	-2.92	-3.10	-3.04	-3.03	-3.10	-3.37	-3.49	-3.34	-3.42	-3.42	-4.09	-3.95
	(3.14)	(2.82)	(2.86)	(2.64)	(2.81)	(2.80)	(2.56)	(2.69)	(2.80)	(2.90)	(2.72)	(2.84)	(2.88)	(3.02)	(3.03)
16	-3.05	-3.18	-3.18	-3.27	-3.45	-3.36	-3.50	-3.40	-3.80	-3.73	-3.75	-3.77	-3.71	-4.45	-4.28
	(3.15)	(3.09)	(2.93)	(3.08)	(2.83)	(3.03)	(3.19)	(2.97)	(3.03)	(2.91)	(3.07)	(3.06)	(2.98)	(3.20)	(3.31)
17	-3.51	-3.61	-3.62	-3.59	-3.54	-3.84	-3.71	-3.81	-4.23	-4.09	-4.09	-3.93	-3.77	-4.59	-4.85
	(3.39)	(3.34)	(3.29)	(3.37)	(3.20)	(3.35)	(3.36)	(3.34)	(3.42)	(3.27)	(3.22)	(3.13)	(3.17)	(3.42)	(3.37)
18	-3.80	-3.72	-3.68	-4.07	-4.06	-4.06	-4.01	-3.72	-4.42	-4.58	-4.31	-4.27	-4.24	-4.39	-5.18
	(3.56)	(3.40)	(3.44)	(3.68)	(3.61)	(3.46)	(3.24)	(3.50)	(3.48)	(3.59)	(3.50)	(3.41)	(3.65)	(3.45)	(3.71)

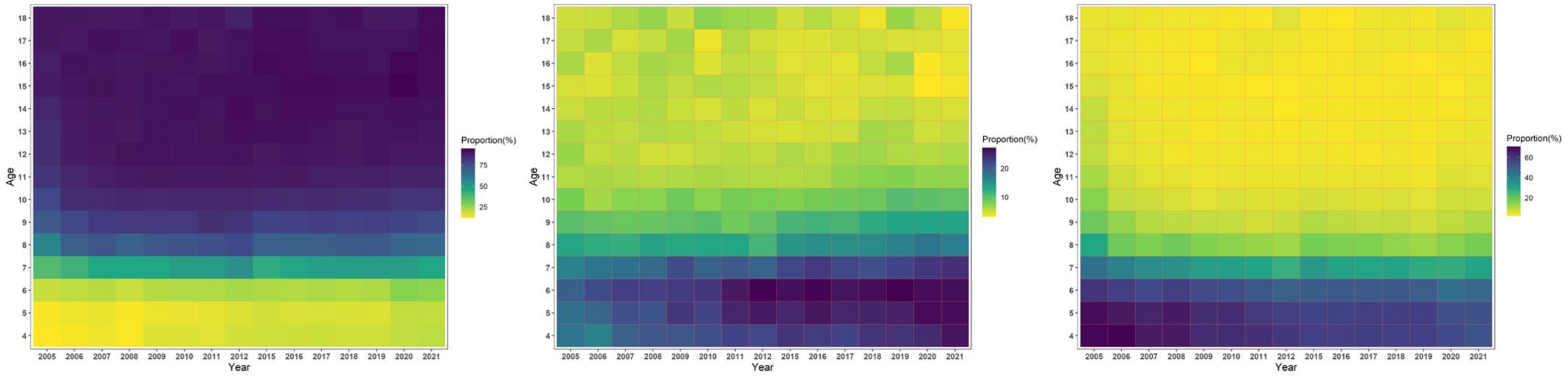

A. Myopia B. Pre-myopia C. Hyperopia

Figure S1. Proportion of myopia (a), pre-myopia (b) and hyperopia (c) in children aged 4-18 years from 2005 to 2021

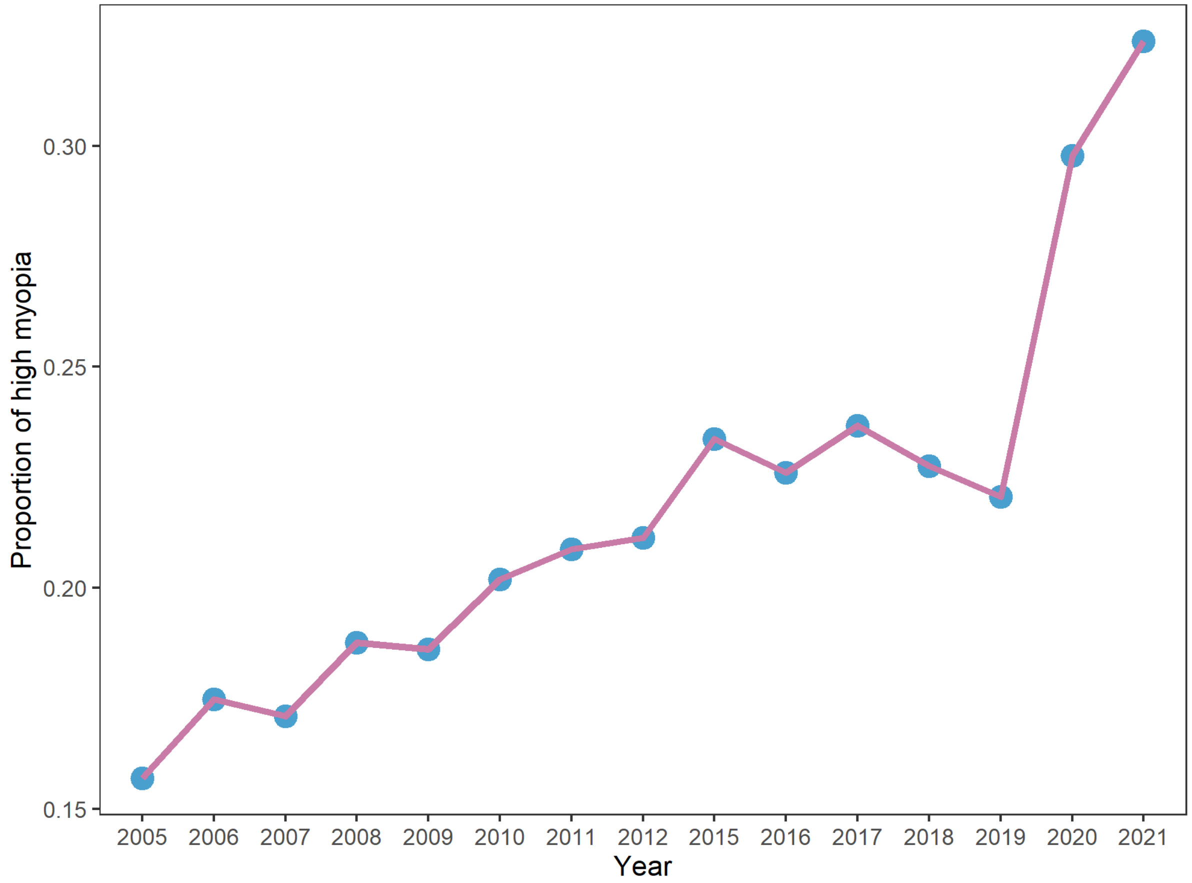

Figure S2. Proportion of high myopia in 16-18-year-old children from 2005 to 2021
